# Supplementary figures and images for: AdipoRon Alleviates Liver Injury by Protecting Hepatocytes from Mitochondrial Damage Caused by Ionizing Radiation
Source: Int J Mol Sci. 2024 Oct 20;25(20):11277. doi: 10.3390/ijms252011277 (PMC11508598; doi:10.3390/ijms252011277)

Figure. S1

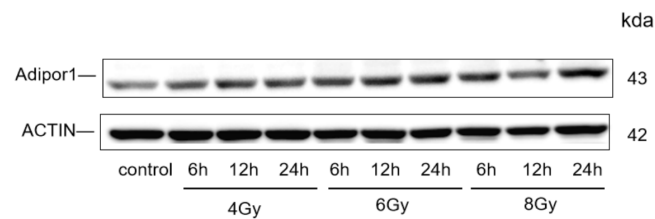

Supplement: Supplementary file 1 [file ijms-25-11277-s001.zip › ijms-3239787-supplementary.pdf]
